# Functional improvement in individuals with chronic spinal cord injury treated with 4-aminopyridine: A systematic review

**DOI:** 10.3389/fneur.2022.1034730

**Published:** 2022-11-29

**Authors:** Martin Paredes-Cruz, Israel Grijalva, Yoscelina Estrella Martínez-López, Gabriel Guizar-Sahagún, Eloisa Colín-Ramírez, David Rojano-Mejía

**Affiliations:** ^1^Epidemiology and Health Services Research Unit, Siglo XXI National Medical Center, Mexican Social Security Institute (IMSS), Mexico City, Mexico; ^2^Doctoral Program in Medical, Dental and Health Sciences, National Autonomous University of Mexico (UNAM), Mexico City, Mexico; ^3^Neurological Diseases Research Unit, Siglo XXI National Medical Center, Specialty Hospital of the Highly Specialized Medical Units (UMAE), Mexican Social Security Institute (IMSS), Mexico City, Mexico; ^4^School of Sport Sciences, Universidad Anahuac Mexico, Huixquilucan, Mexico; ^5^Health Research Coordination Department, Siglo XXI National Medical Center, Mexico City, Mexico

**Keywords:** 4-aminopyridine, spinal cord injury, functional improvement, efficacy, motor function, sensitive functions, sphincter control, clinical trials

## Abstract

**Study design:**

Systematic review.

**Objective:**

To provide current evidence on the efficacy of 4-aminopyridine (4-AP) to bring about functional improvement in individuals with chronic traumatic spinal cord injury (SCI).

**Methods:**

The Medline (PubMed), Web of Science and SCOPUS databases were systematically searched for relevant articles on the efficacy of 4-AP to treat SCI, from the dates such articles were first published until May 2022. Full-text versions of all the articles selected were examined independently by two reviewers. Methodological quality was rated using the Modified Jadad Scale, and risk of bias was assessed with the RoB-2 test. Data extracted included human models/types, PRISMA assessment protocols, and the results of each study. Descriptive syntheses are provided.

**Results:**

In total, 28 articles were initially identified, 10 of which were included after screening. Most of the studies reviewed reported some degree of patient improvement in one or more of the following parameters: motor, sensitivity and sexual function, sphincter control, spasticity, ability to function independently, quality of life, central motor conduction, pain, and pulmonary function.

**Conclusions:**

This review confirms the efficacy of 4-AP in improving several conditions resulting from SCI but further research on this topic is warranted. Additional randomized clinical trials with 4-AP involving larger sample sizes are needed, as are consistent outcome measures in order to obtain adequate data for analysis with a view to enhance treatment benefits.

**Systematic review registration:**

https://www.crd.york.ac.uk/prospero/display_record.php?RecordID=334835, PROSPERO CRD42022334835.

## Introduction

Four-Aminopyridine (4-AP) is a potassium-channel blocker with the ability to promote action potentials along demyelinated axons (1, 2). The 4-AP compound also aids synaptic transmission by enhancing the flow of presynaptic calcium currents, a function secondary to blocking the potassium channel (2, 3).

This drug was approved in 2012 as a treatment to help improve ambulatory functions in adults with multiple sclerosis (2). Because of its mechanism of action, 4-AP may also be useful to treat alterations resulting from other neurological conditions such as spinal cord injuries (SCIs) (4, 5).

Less than half of traumatic SCIs involve a completely transected spinal cord, even when neurological loss results in a clinically complete injury (6–8). Similarly, magnetic resonance imaging of people with complete injuries has yielded evidence of spinal cord continuity (8). The extent of SCIs depends on the severity of the primary mechanical traumatic event, as well as on the cascade of subsequent secondary events (7). Nonetheless, nerve fibers crossing the epicenter of the lesion often remain intact (9). Accordingly, pharmacological compounds (such as 4-AP) that enhance electrical conduction in surviving axons have been used to improve the condition of the neural pathways that underly locomotor control. This has led to functional benefits for individuals after injury (9, 10).

Various authors have identified functional improvement in patients with spinal cord injuries, although methodologies and outcomes vary and point to benefits in different areas—mainly motor function, sensitivity, sexual function, sphincter control, spasticity and functional independence—depending on the specific purpose of each study.

In light of the above, we carried out a systematic review to assess the efficacy of 4-AP to improve functionality in traumatic SCI patients.

## Methods

### Literature search strategy

**Figure 1:**
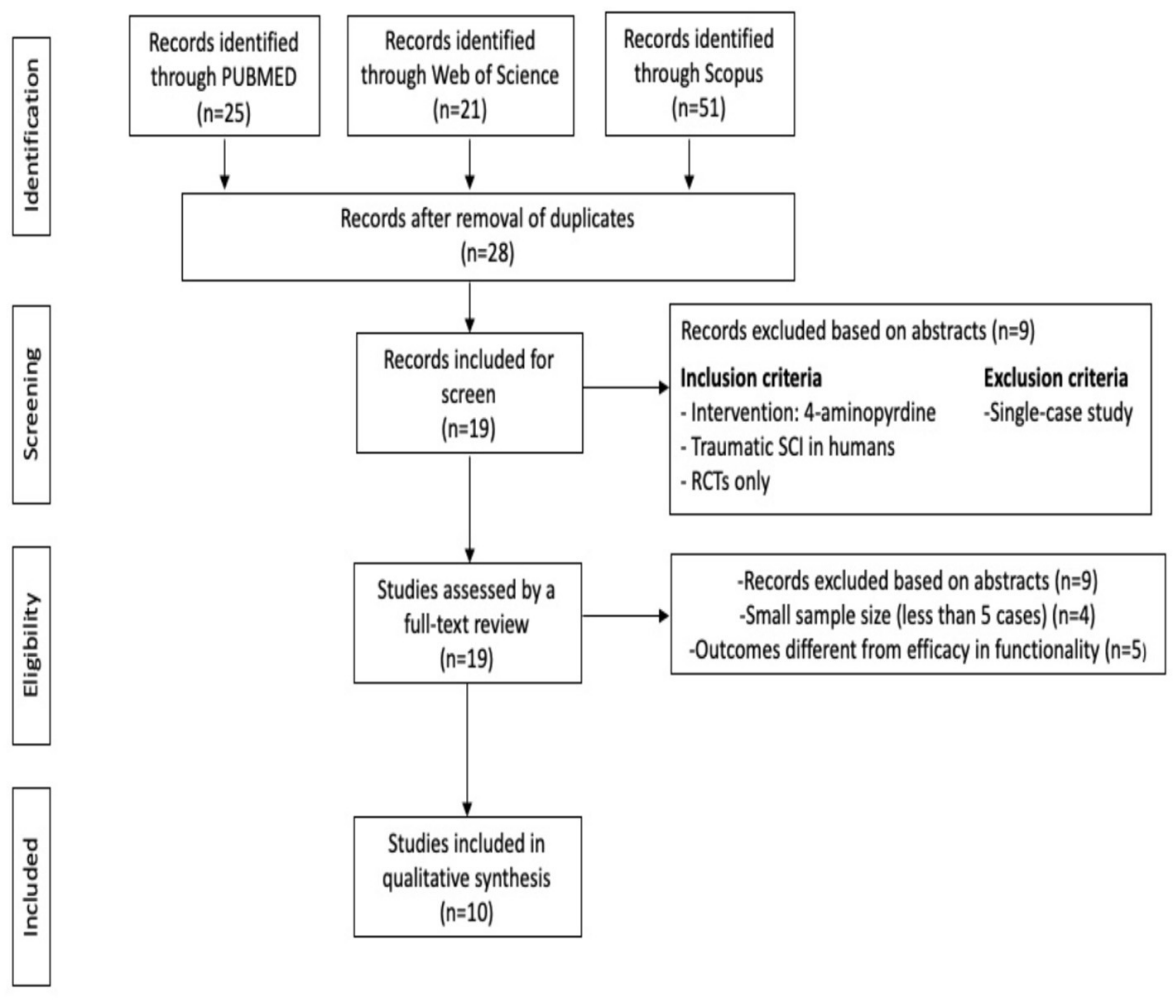
PRISMA flow diagram describing the screening and review process (11).

This systematic review followed the guidelines in Preferred Reporting Items for Systematic Reviews and Meta-Analyses (PRISMA) (11), while the study protocol was registered with PROSPERO (CRD42022334835). We used the following databases to identify studies relevant for an electronic search in current literature: PubMed (MEDLINE), Web of Science and Scopus, until May 26, 2022. In this paper we have used various combinations of the following terms: spinal cord injuries (SCI), 4-aminopyridine (4-AP), patients, and humans. Our search was limited to the following kinds of documents: articles, human clinical trials and literature in English. Our investigation included original research studies investigating the efficacy of 4-AP for treating individuals with traumatic SCI (Figure 1).

### Selection criteria

Our study defined eligibility criteria according to PICOS variables: Population (P), Intervention (I), Comparator (C), Outcome (O) and Study Design (S).

*Population*. Individuals diagnosed with SCI (either complete or incomplete) who had been given 4-AP in a clinical trial as an intervention to manage or treat their condition.

*Intervention*. Studies using 4-AP administered both orally and intravenously were included, and all dosage levels were considered.

*Comparator*. Individuals received either 4-AP or a placebo as comparator.

*Results*. Included are studies that reported the effect of 4-AP in humans in the context of any long-term quantitative or qualitative clinical outcome. Results included scores for motor and sensory functions, functional independence, sphincter control, sexual function, quality of life, pain, spasticity and central motor conduction. Also included are data on secondary outcomes, like adverse reactions, as indicators of safety.

*Study design*. Includes primary research studies and randomized clinical trials (RCTs) only, and excludes reviews, pilot studies, prospective studies, retrospective studies and case series, single-case studies, editorial reports, and protocols.

### Studies selected and data extracted

We identified articles using the search strategy described above. Based on titles and abstracts, we then eliminated duplicate results and included or excluded articles according to the PICOS criteria indicated. We reexamined the articles, scrutinized their full text and assessed their methodological quality before including them in our systematic review. Next, we rated the quality of the clinical trials according to the Modified Jadad Scale (12). Descriptive syntheses of the findings of all studies are provided in the text and tables below (Tables 1, 2).

**Table 1:** Clinical trial quality evaluation using the Modified Jadad Scale.

**Items**	**Hansebout 1993 (14)**	**Potter 1998 (15)**	**Segal 1999 (16)**	**Wolfe 2001 (17)**	**van der Bruggen 2001 (18)**	**Grijalva 2003 (19)**	**DeForge 2004 (20)**	**Cardenas 2007 (21)**	**Grijalva 2010 (22)**	**Cardenas 2014 (23)**
Would you describe this study as random?	X	X	X	X	X	X	X	X	X	X
Would you describe this study as double-blinded?	X	X	X	X	X	X	X	X	X	X
Are dropouts and exclusions from the study described?	–	X	X	–	X	X	X	X	X	X
Is the random-assignment method adequate?	X	X	X	X	X	X	X	X	X	X
Is the masking method adequate?	–	X	X	X	X	X	X	X	X	X
Is the frequency of adverse events clearly described?	X	X	X	–	X	X	X	X	X	X
Are eligibility criteria clearly defined?	X	X	X	X	X	X	X	X	X	X
Is the method for obtaining the sample size described?	–	–	–	–	–	–	X	X	–	–
Total score	5	7	7	5	7	7	8	8	7	7

**Table 2:** Main characteristics of randomized control trials assessing the effects of 4-aminopyridine on the treatment of spinal cord injury.

**Efficacy of 4-aminopirydine (4-AP)**								
**Author** **Country** **Year**	**Participants**	**Sample size**	**Intervention implemented/control**	**Number of participants (basal, final)**	**Treatment duration**	**Aims/outcomes**	**Significance difference between groups**	**Safety**
Hansebout et al. (14) Canada 1993	Male and female Intervention: 18 ± 65 years old, with spinal cord injury (SCI) including cases of quadriplegia, quadriparesis, paraplegia and paraparesis	8	**Intervention:** 4-AP intravenous solution, with dose escalated from 18.0 to 33.5 mg/day **Control:** Placebo	**Intervention:** 8 **Control:** 8	Two weeks	**Primary:** To improve neurological status (motor and sensory control) as well as functionality below the injury, and to reduce chronic pain and spasticity after drug administration	**Yes:** Administration of the drug was associated with significant temporary neurological improvement in 5/6 of individuals with incomplete SCI. Improvements in neurological status following drug administration included increased motor control and sensory functionality below the injury, as well as reduction in chronic pain and spasticity.	The most frequently detected side effect of the drug was discomfort in the arm in which the drug was infused. Two of the participants reported severe burning and aching in the arm; both also experienced heightened anxiety accompanied by short, alternating episodes of perspiring and shivering toward the end of the infusion period. Two individuals reported a feeling of light-headedness toward the end of the infusion period. Two reported delayed burning sensations in areas of skin below the level of injury, lasting for 1–2 h during the night after the infusion.
Potter et al. (15) Canada 1998	Female 21–65 years old: medical diagnosis of incomplete tetraplegia/paraplegia made >2 years prior to the study, neurological level of injury C4-T12, medically stable and able to breathe independently, and stable neurological deficits for >60 days prior to the study	26	**Intervention:** Sustained-release fampridine (fampridine-SR), with dose escalated from 12.5 to 17.5 mg BID **Control:** Placebo	**Intervention:** 29, 26 **Control:** 29, 26	2 weeks	**Primary:** To improve motor and sensory index scores, sphincter control and sexual variables, as well as to reduce pain and spasticity	**Yes:** Participants reported significant benefits from fampridine-SR over placebo as regards individual satisfaction (*p* < 0.05) and quality-of-life scores (*p* < 0.01). Sensory scores (*p* < 0.01), including pinprick (*p* = 0.059) and light touch (*p* = 0.058), as well as motor scores (adjusted to reflect only paretic segments) (*p* < 0.01), all yielded evidence of benefits from fampridine-SR over placebo. The Modified Ashworth scores for spasticity dropped significantly (*p* < 0.05) when individuals received fampridine-SR.	Assessment of the temperature, pulse and systolic and diastolic blood pressure showed no significant differences across the stages of the study or within groups. Fampridine-SR induced no seizures. There were reports of mild and transient giddiness or lightheadedness (*n* = 5) at the onset of drug administration.
Segal et al. (16) USA 1999	Male and female Outpatients suffering from traumatic SCI (14 tetraplegic and 7 paraplegic) for ≥ 2 years	21	**Intervention:** 4-AP oral dose: 30 mg/day (high dose), blinded **Intervention:** 4-AP oral dose: 30 mg/day (high dose), unblinded **Control:** 4-AP oral dose: 6 mg/day (low dose)	**Intervention:** 6, 6 **Intervention:** 10, 10 **Control:** 5, 4	3 months	**Primary:** To determine the effects of long-term administration of 4-AP on sensorimotor functions in humans with longstanding SCI **Secondary:** To assess spasticity based on the Modified Ashworth Scale	**Yes:** Composite motor and sensory scores showed statistically significant increases at 3 months. Maximal expiratory pressure, maximal inspiratory pressure, forced vital capacity, and forced expiratory volume in 1 second indicated clinically meaningful and/or statistically significant increases among participants receiving 4-AP 30 mg/day. These individuals also exhibited significant decreases in spasticity (Modified Ashworth Scale).	Neither clinically significant adverse effects nor measurable toxicity occurred. Nervousness, giddiness or dizziness, and gastrointestinal upset manifesting as mild abdominal cramping or nausea were the most frequent side effects.
Wolfe et al. (17) Canada 2001	Male and female The majority of participants suffered injuries as a result of trauma, and presented myelopathy due to transverse myelitis, occlusion of the anterior spinal artery, cervical spinal abscess and cervical spinal stenosis.	25	**Intervention:** 4-AP oral dose: 10 mg/day **Control:** Placebo	**Intervention:** 25 **Control:** 25	2 weeks	**Primary:** To reduce central motor conduction time (CMCT) and determine whether motor-evoked potentials (MEPs) can be recorded from paretic muscles	**Yes:** The principal finding was that 4-AP lowered the stimulation threshold, increased the amplitude, and reduced the latency of MEPs in all muscles tested, including those that were unimpaired, but did not alter the measures of the peripheral nervous system. These 4-AP–induced changes in MEPs were significantly greater than those seen with placebo (*p* = 0.05).	
Van der Bruggen et al. (18) Netherlands 2001	Male and female 4-AP: 46 ± 13.9 years old. Placebo: 42.7 ± 14 years old	19	**Intervention:** 4-AP oral dose: 5 mg/day increased to a daily maximum of 0.5 mg/kg body weight **Control:** Placebo	**Intervention:** 10, 9 **Control:** 10, 10	1 month	**Primary:** To determine the efficacy of 4-AP on functional status, gait speed and vibration perception in individuals with chronic, incomplete SCI	**Yes:** Only in functional status, significant inter-group differences were observed after the wash-out period (t4). The differences were in favor of Group 1 and related to overall health (p=0.04) and social activities (p=0.04).	In the treatment group, 4 individuals registered mild and transient side effects including giddiness and headache as well as feelings such as “having the flu.” In the placebo control group, 5 individuals reported mild complaints of headache, dizziness, light- headedness and feeling sick.
Grijalva et al. (19) Mexico 2003	Male and female 4-AP: 34 ± 8.4 years old Placebo: 33 ± 7.9 years old	25	**Intervention:** 4-AP oral dose: 5 mg/day, escalated by 5 mg/week to a maximum of 30 mg/day **Control:** Placebo	**Intervention:** 14, 13 **Control:** 13, 12	3 months	**Primary:** To study the efficacy and safety of 4-AP **Secondary:** To document sensorimotor changes after discontinuation of the drug in individuals with long-term SCI	**Primary, yes:** Success was observed in 25/36 of areas for the 4-AP group vs. only 18/39 of areas for the placebo group (*p* = 0.042). **Secondary, yes:** 8/12 of participants preserved function of sensation (*p* = 0.032). Sensation improved by 49% on average compared with scores at the end of 4-AP intake. 10/12 of individuals experienced persistent improvement in independence (*p* = 0.042)	Fourteen individuals treated with 4-AP experienced 26 probable adverse effects, of which only 3 were found to be definitively associated with 4-AP. Adverse effects appeared from the start of weekly dose increases and from 15 to 45 min after taking 4-AP. They generally resolved within 1–4 h after taking 4-AP and disappeared within 3–5 days of continued treatment. Dry mouth, dizziness and gastritis began with 4-AP 5 or 10 mg/day; oral and peripheral paresthesia appeared only with 4-AP 30 mg/day; no epileptic seizures occurred.
DeForge et al. (20) Canada 2004	Male and female AP: 40.13 ± 13.63 years old Placebo: 40.13 ± 13.63 years old 24–57 years old	14	**Intervention:** 4-AP oral dose: 40 mg/day **Control:** Placebo	**Intervention:** 15 **Placebo:** 14	2 weeks	**Primary:** To determine the efficacy of 4-AP in improving lower-limb muscle strength and biomechanical gait patterns of chronic SCI	**No**	
Cardenas et al. (21) USA 2007	Male and female 4-AP (25 mg): 44 (12–66) years old 4-AP (40 mg): 42 (21–67) years old Placebo: 38 (19–61) years old	71	**Intervention 1:** 4-AP oral dose: 25 mg/day **Intervention 2:** 4-AP oral dose: 40 mg/day **Control:** Placebo	**Intervention 1:** 30, 26 **Intervention 2:** 30, 17 **Control:** 31, 28	2 months	**Primary:** To determine the safety and efficacy of fampridine-SR in individuals with chronic SCI **Secondary**: To determine the International Index of Erectile Function (IIEF-15) values and assess spasticity (Modified Ashworth Scale) in the intervention	**Primary, yes:** Intervention groups 1 and 2 experienced an increase in the number of days with bowel movements compared to the placebo group (*p* = 0.02 and *p* = 0.01, respectively). Less frequent bladder accidents were registered in the group treated with fampridine 25 mg twice a day (BID) compared to the placebo group. **Secondary, yes:** Subjects in the fampridine 25 mg BID group showed a statistically significant improvement in SGI scores compared with those in the placebo group (p < 0.02). Erection frequency and firmness, ability to maintain erections and levels of sexual desire showed greater improvement in the fampridine groups than in the placebo group (*p* = 0.02). The Ashworth scores showed a strong trend toward improvement in the 25 mg BID group compared to the placebo group (*p* < 0.04).	Most treatment emergent adverse events (TEAEs) were mild to moderate in severity and were transient. As noted below in the subsequent Cardenas RCT, a total of 16 individuals were discontinued due to adverse events: 2 from the placebo, 3 from the 25 mg BID and 11 from the 40 mg BID group. The TEAEs most frequently associated with discontinuation were dizziness (8%), insomnia (4%) and nausea (3%). Only one serious adverse event (SAE), a seizure in an individual with a history of traumatic brain injury, was considered probably related to the study drug. The person was in the 40 mg BID group and had been taking study medication for ~7 weeks. Another individual, also in the higher-dose group, developed gastrointestinal bleeding, assessed as having a possible relationship to the study drug.
Grijalva et al. (22) Mexico 2010	Male and female 4-AP: 29 ± 6.21 years old Placebo: 29 ± 6.21 years old. 20–40 years old	14	**Intervention 1:** 4-AP oral dose: increased gradually from 5 mg/week to 30 mg/day; for long-term treatment, dose was escalated from 10 mg/day to 1 mg/kg/day **Control:** Placebo	**Intervention 1:** 9, 9 **Control:** 4, 4	3 months	**Primary:** To test the functional effect of high doses of 4-AP on individuals with chronic complete SCI with cord continuity at the site of injury demonstrated by magnetic resonance imaging	**No:** No significant changes were found in either the clinical or the electrophysiological evaluations. **In the second phase:** 7/12 of individuals with higher clinical scores also showed improvement in the somatosensory evoked potentials, including a better definition of the radiculo-medullary component and higher cortical wave voltage; 3/12 of these individuals were able to walk with assistance; 1/12 changed from a complete Asia Impairment Scale (AIS) A to an incomplete AIS B SCI classification; 5/12 had sensation as well as control of bladder and anal sphincters; and 4/9 of male participants had a psychogenic erection.	Individuals who received 4-AP presented varying degrees of toxicity, with the most frequent being neuropsychiatric alterations such as paresthesia, spasms, insomnia, amnesia, seizures, alterations in personality, etc. The seizure experienced by the person mentioned in the previous section was found to be related to the use of 4-AP; it disappeared when the drug was discontinued.
Cardenas et al. (23) USA/ Canada 2014	Male and female Study 1 4-AP: 41± 12.1 years old Placebo: 40 ± 13.1 years old Study 2 4-AP: 41.3± 11.8 years old Placebo: 40.5 ± 12.3 years old	Study 1: 212 Study 2: 203	**Study 1 intervention:** Fampridine-SR oral dose: 25 mg BID **Control:** Placebo **Study 2 Intervention:** Fampridine-SR oral dose: 25 mg BID **Control:** placebo	**Study 1** **Intervention:** 114, 114 **Control:** 99, 98 **Study 2** **Intervention:** 104, 104 **Control:** 100, 100	4 months	**Primary:** To evaluate the efficacy and safety of fampridine-SR tablets in individuals with chronic SCI	**Study 1, yes:** The only significant between-treatment differences were a slightly greater improvement among men treated with fampridine-SR in two IIEF domains: erectile function (*p* = 0.016) and orgasmic function (*p* = 0.032). **Study 2, yes:** A significant between-treatment difference occurred in the Upper Extremity Subscale. Furthermore, a significantly greater increase in the number of bowel movements was registered among individuals treated with fampridine-SR vs. those treated with a placebo (*p* < 0.006).	TEAEs were generally of mild or moderate severity. Within the fampridine-SR group in Study 1, the most common TEAEs leading to discontinuation were dizziness and hypertonia in 6/98 of individuals, as well as insomnia and asthenia in 3/114. Similar proportions and reasons for TEAE-related discontinuation were reported in Study 2: 3/100 and 16/103 of individuals in the placebo and fampridine-SR groups, respectively, experienced dizziness; 4/103 hypertonia; and 3/103 paresthesia, with these being the most common TEAEs leading to discontinuation for individuals treated with fampridine-SR.

After critically evaluating the articles, two reviewers (MPC, YEML) screened the abstracts and full texts, extracted data and utilized a spreadsheet to record the information. Data extraction focused on: author, country, year, inclusion criteria, sample size, intervention, number of participants (at baseline and at the end of the study), duration of treatment, study objectives, as well as significant differences between groups. The team resolved any discrepancies regarding data extraction through discussion.

### Assessment of risk of bias in selected trials

In order to assess risk of bias (RoB), the reports were reviewed independently by two reviewers (MPC, YEML) using RoB-2 (Risk of Bias in Randomized Studies to Assess Human-Centered Studies) (13). Through discussion, the team resolved any disagreements over the RoB assessment.

## Results

### Study selection

This study identified a total of 28 abstracts. After eliminating duplicates and selecting abstracts, 19 articles were considered eligible for full-text evaluation. Of these, 10 were included in the final synthesis as shown in Figure 1 (14–23). Tables 1, 2 provide general descriptions.

### Location and study design

The studies took place in Canada (14, 15, 17, 20), the United States (16, 21, 23), Mexico (19, 22) and the Netherlands (18). Median sample size was 23 participants. All studies involved RCTs.

### Risk of bias in the selected studies

**Figure 2:**
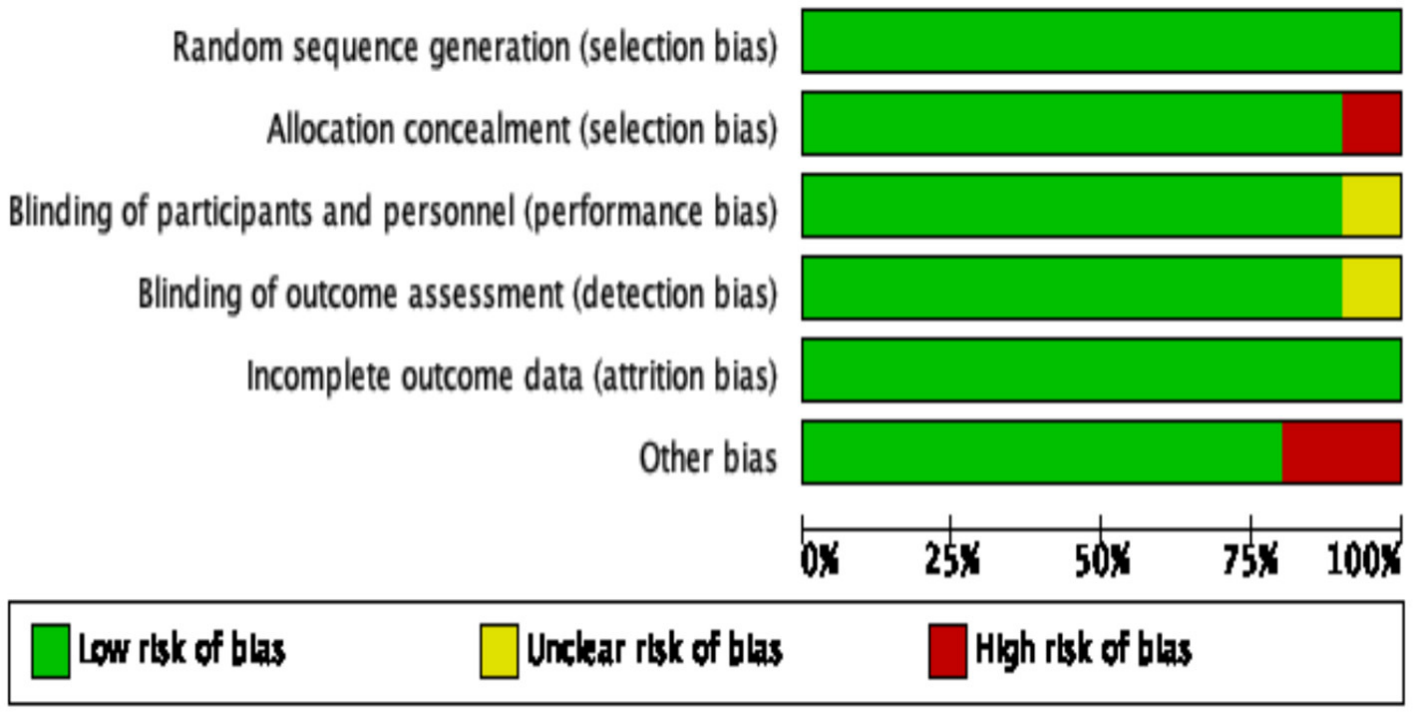
Graphic representation of risk of bias in randomized control trials assessing the effects of 4-aminopyridine on the management of spinal cord injury.

**Figure 3:**
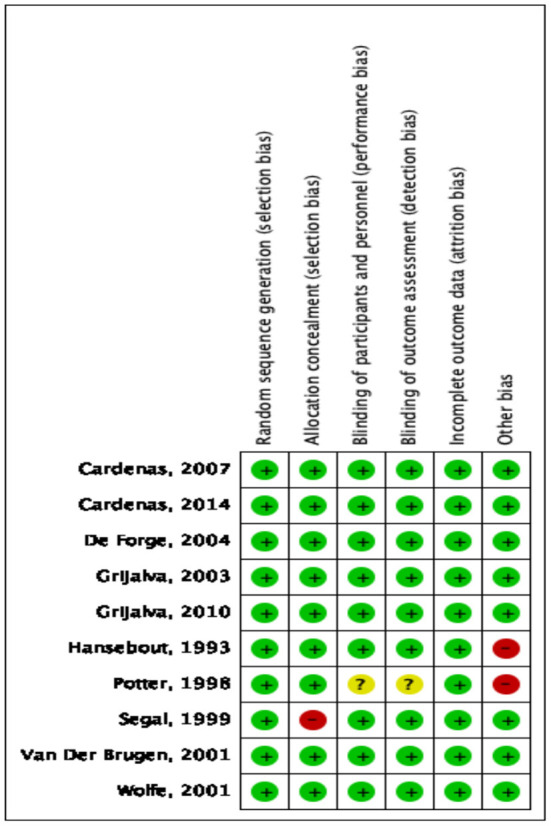
Risk-of-bias summary for randomized control trials assessing the effects of 4-aminopyridine on the management of spinal cord injury.

The results of our bias risk assessment for each trial are shown in Figures 2, 3. All trials were rated low risk of bias for random sequence generation. Nine were classified as low risk of bias for allocation concealment, participant and personnel blinding, as well as for outcome and incomplete results assessment blinding. Two trials were rated as high risk for other biases, primarily because their sample size was small.

### Efficacy of 4-aminopyridine in individuals with traumatic spinal cord injury

Five of the 10 studies selected used the ASIA Impairment Scale (AIS) and focused on neurological status (motor and sensory control). Improvement was found in four of these variables (14–16, 19) among individuals taking 4-AP as opposed to a placebo. Five studies used the Modified Ashworth Scale to assess spasticity; three of them (14–16) reported improvement. Five studies assessed sexual function; three used the International Index of Erectile Function (IIEF) and two, a clinical interview questionnaire. Four of these five studies indicated improvement (20–23). Three of the four studies that assessed sphincter control found improvement (21–23). Two of the four studies evaluated functional independence using the Spinal Cord Independence Measure (SCIM), while one used the Functional Independence Scale (FIM), and another, the WONCA/COOP Functional Health Assessment Scale. Three of the four studies evaluating functional independence demonstrated improvement (15, 18, 19). Two studies assessed pain with the McGill Pain Questionnaire but only one reported improvement (14). None of the studies that focused on gait speed and vibration perception showed any improvement (18, 20). Each of the following functions was evaluated using a single test for each. All of them identified benefits: central motor conduction was assessed with the Motor Evoked Potentials (MEP) test; quality of life with the 7-point Terrible-Enchanted Scale; and pulmonary function, with an appropriate lung capacity test (Table 2).

### Safety

Of the 10 articles included in this review, eight secondarily evaluated 4-AP safety and identified mild-to-moderate adverse events; few articles reported serious events (Table 2).

## Discussion

This systematic review examined existing literature on the efficacy of 4-aminopyridine (4-AP) as a treatment for spinal cord injury (SCI). Ten studies were included of which three yielded insufficient results to pool with the findings of other research. The remaining seven studies provided evidence that in various respects 4-AP improved functionality in individuals with traumatic SCI.

In the evidence supported by our systematic review, we observed that efficacy of 4-AP to improve function mainly depends on two circumstances: first, that the tract is preserved and the extent to which it is myelinated (24–30); and second, the main objective of each study. Therefore, it is to be expected that patients will not improve in every way. Significant improvements in neurological status–specifically in motor and sensory functions, functional independence, sphincter control and sexual function–were observed in both men and women, along with improvements in quality of life, pain, spasticity and central motor conduction. Drug intake ranged from a maximum dose of 10 mg per day to 1.45 mg per kilogram of body weight per day. The greatest benefits resulted from higher doses. Administration periods ranged from 2 weeks to 1 year in open-label clinical trials. The greatest changes were identified in individuals with incomplete SCI compared to patients with complete SCI.

There are no RCTs on medium or long-term treatment of spinal cord injury patients with 4-AP. Nevertheless, two Phase III clinical trials of multiple sclerosis evaluated open long-term doses of 10 mg of 4-AP twice a day (20 mg/day) for a maximum period of 5 years. These trials proved that improvements were maintained during long term use and adverse events were similar to those previously reported in prior studies (31, 32). On the other hand, in the experience of our team (still unpublished data) treatment was given to openly enrolled patients for a long-term ranging from 3 months to 3 years during which 4-AP was safe. It appears, and the team considers, that the presence or absence of adverse events depends on personal susceptibility because some patients presented mild adverse events at low doses, while others at high doses presented none. Now, in terms of severe adverse events, convulsions are the events of greatest concern, but in this case it was determined that doses<40 mg/day were safe and no severe adverse events occurred, whereas at doses greater that 40 mg/day the risk of convulsions increased (22, 23). Most of these studies assessed 4-AP safety and identified mild-to-moderate adverse events that would not impede treatment continuity, as well as few serious events, such that 4-AP was considered safe even at high doses (1.45 mg/kg/day) (22). As mentioned before, one of the alleged mechanisms of action of 4-AP is that it increases action potential conduction in demyelinated fibers, thus improving their strength. It is likewise believed that 4-AP increases neuronal excitability and potentiates synaptic transmission (24–29). For all of this, the action and toxicity of this drug could be due to either one of these two mechanisms, however optimal dose to maximize the risk-benefit ratio appears to depend on the amount of axons preserved after the injury, as well as their degree of demyelination.

Unfortunately, not all of the articles included in our review assessed the same outcomes, although 9 of the 10 articles included in this study proved the efficacy of 4-aminopyridine to improve function, particularly motor and sensitivity function (14–16, 19), sexual function (20–23), sphincter control (21–23), functional independence (15, 18, 19), and spasticity (14–16). Although these published articles did not express patient preferences regarding their expectations for improvement, our group's experience indicates that patients assign the greatest importance to functional independence mainly because it involves sphincter control and mobility. Therefore, it will be important to evaluate these two results variables in experimental studies. In terms of these considerations, 7 of the 10 articles included in our own study proved 4-AP efficacy in these variables: 3 showed efficacy in sphincter control (21–23), and 4 in motor function (14–16, 19).

Although the main result of this study demonstrated the efficacy of 4-AP, study variables are heterogeneous and therefore made it difficult to perform a meta-analysis. We recommend that future studies conduct uniform and comprehensive assessments employing the same outcome variables. While some of the studies analyzed demonstrated no statistically significant differences, substantive clinical benefits were achieved. These included walking with the help of devices, enhanced sensation, improved bladder and anal sphincter control, psychogenic erections in men, and improvements in daily living, which provided individuals greater functional independence.

The results were more encouraging when specific functions–bladder and anal sphincter control, quality of life and functional Independence– were comprehensively evaluated along with sensory and motor functions. As noted by Cardenas et al. (21), even minimal improvements in bladder control and sexual function were enormously significant in the daily lives of individuals with chronic SCI.

Despite improvements in gait brought about by administering 4-AP to individuals with multiple sclerosis, clinically significant, long-lasting effects appeared soon after initiation of treatment, yet disappeared shortly after drug withdrawal (31–34). In the case of individuals with SCI, however, such benefits appeared to last even after treatment had ended (14, 16, 19, 22).

Other studies indicated that the effects of 4-AP seemed to differ depending on the selection of “responsive participants,” where different variables were in play: (a) the severity of the injury, as individuals with cervical injury apparently showed greater improvement than those with thoracic or lumbar injury; (b) the type of injury (complete or incomplete AIS classification), SCIs were not uniform and affected ascending and descending tracts in a variety of ways—the recovery of a function depended on the tract affected, so improvement varied in each individual; and (c) the phase of the injury: in the acute phase, preserved axons were demyelinated or insufficiently myelinated and therefore, long-term myelination in the chronic phase could support response to treatment. All of the above factors make it difficult to evaluate the efficacy of any pharmacological intervention among this population (14, 16, 19, 35, 36).

Clinical efficacy of 4-AP is currently still under evaluation *via* randomized controlled clinical trials in pathologies such as multiple sclerosis (NCT01576354), spinal cord injury (NCT03899584, NCT05447676, NCT01621113), Guillain-Barré syndrome (NCT00056810), among others.

### Limitations

Limiting this systematic review to literature in English entailed the risk of language bias in our selection of studies, while the inclusion of studies with heterogeneous results did not allow us to perform a meta-analysis, only a qualitative synthesis.

Varied outcome measures were used in the studies reviewed, which made it difficult to compare results. We found considerable variation among studies in terms of route, dose, and/or duration of treatment with 4-AP. All these factors were also considered limitations when evaluating the efficacy of this drug.

## Conclusion

There is a dearth of literature on the efficacy and safety of 4-AP in treating individuals with traumatic SCI. Although this systematic review provides information showing that 4-AP is an effective treatment for improving some functions after chronic SCI, further randomized clinical trials with 4-AP involving larger sample sizes are needed. Future research should use uniform outcome measures to allow adequate data acquisition and analysis.

## Data availability statement

The original contributions presented in the study are included in the article/supplementary material, further inquiries can be directed to the corresponding authors.

## Author contributions

DR-M and IG supervised the findings. MP-C and YM-L contributed to data collection, extraction, and analysis and developed the theory. IG, EC-R, GG-S, and DR-M made critical contributions and final approval of the manuscript. All authors discussed the results and contributed to the final manuscript.

## Conflict of interest

The authors declare that the research was conducted in the absence of any commercial or financial relationships that could be construed as a potential conflict of interest.

## Publisher's note

All claims expressed in this article are solely those of the authors and do not necessarily represent those of their affiliated organizations, or those of the publisher, the editors and the reviewers. Any product that may be evaluated in this article, or claim that may be made by its manufacturer, is not guaranteed or endorsed by the publisher.
